# Quantized Microcavity Polariton Lasing Based on InGaN Localized Excitons

**DOI:** 10.3390/nano14141197

**Published:** 2024-07-14

**Authors:** Huying Zheng, Runchen Wang, Xuebing Gong, Junxing Dong, Lisheng Wang, Jingzhuo Wang, Yifan Zhang, Yan Shen, Huanjun Chen, Baijun Zhang, Hai Zhu

**Affiliations:** 1 State Key Laboratory of Optoelectronic Materials and Technologies, School of Physics, Sun Yat-sen University, Guangzhou 510275, China; zhenghy37@mail2.sysu.edu.cn (H.Z.); wangrch7@mail2.sysu.edu.cn (R.W.); gongxb@mail2.sysu.edu.cn (X.G.); dongjx3@mail2.sysu.edu.cn (J.D.); wanglsh23@mail2.sysu.edu.cn (L.W.); wangjzh23@mail2.sysu.edu.cn (J.W.); zhangyf93@mail2.sysu.edu.cn (Y.Z.); 2 State Key Laboratory of Optoelectronic Materials and Technologies, School of Electronics and Information Technology, Sun Yat-sen University, Guangzhou 510275, China; chenhj8@mail.sysu.edu.cn

**Keywords:** microcavity, localized exciton, polariton, quantization

## Abstract

Exciton–polaritons, which are bosonic quasiparticles with an extremely low mass, play a key role in understanding macroscopic quantum effects related to Bose–Einstein condensation (BEC) in solid-state systems. The study of trapped polaritons in a potential well provides an ideal platform for manipulating polariton condensates, enabling polariton lasing with specific formation in k-space. Here, we realize quantized microcavity polariton lasing in simple harmonic oscillator (SHO) states based on spatial localized excitons in InGaN/GaN quantum wells (QWs). Benefiting from the high exciton binding energy (90 meV) and large oscillator strength of the localized exciton, room-temperature (RT) polaritons with large Rabi splitting (61 meV) are obtained in a strongly coupled microcavity. The manipulation of polariton condensates is performed through a parabolic potential well created by optical pump control. Under the confinement situation, trapped polaritons are controlled to be distributed in the selected quantized energy sublevels of the SHO state. The maximum energy spacing of 11.3 meV is observed in the SHO sublevels, indicating the robust polariton trapping of the parabolic potential well. Coherent quantized polariton lasing is achieved in the ground state of the SHO state and the coherence property of the lasing is analyzed through the measurements of spatial interference patterns and g^(2)^(τ). Our results offer a feasible route to explore the manipulation of macroscopic quantum coherent states and to fabricate novel polariton devices towards room-temperature operations.

## 1. Introduction 

Exciton–polaritons, hybrid bosonic quasiparticles, arise from the strong coupling of excitons to microcavity photons [1]. Due to their small effective mass (nine orders of magnitude smaller than that of an atom), the Bose–Einstein condensation (BEC) of polaritons [2,3] can occur at higher temperatures, which has greatly facilitated the investigation on quantum physical phenomena such as superfluidity [4], bright solitons [5], and quantized vortex states [6], as well as modern optoelectronics [7]. Notably, a coherent spontaneous emission called polariton lasing is obtained through the BEC of polaritons [8]. Since without the requirement of population inversion, polariton lasing exhibits a much lower threshold than traditional photonic lasing [9], presenting potential applications in ultra-low threshold lasers, optical communications, and optical sensing. However, the practical application of polariton lasers, particularly in materials like GaAs [10], is hindered by the low operating temperature condition resulting from their small exciton binding energy.

Wide band gap GaN semiconductors exhibiting large exciton binding energy (*E_b_*) and high oscillator strengths have been regarded as a promising platform for achieving room-temperature polariton lasing [11,12,13]. The exciton binding energy is further enhanced by localized exciton effects in InGaN/GaN quantum wells (QWs) [14], which satisfy the operation of room-temperature polaritons. Nevertheless, thermal broadening at room temperature impedes the realization of polariton lasing with higher coherence. Recently, manipulating polaritons within spatial potential wells has attracted great interest [15]. By designing additional lateral traps within a microcavity, a rich potential energy landscape is possible to create. As a result, the interactions and transport behaviors of polaritons are altered, enabling trapped polariton condensates for highly coherent lasing. Several pioneering works of tailored environments for trapped polariton condensation have been reported [16,17,18,19,20]. Notably, the polaritons trapped by a parabolic potential well occupy a simple harmonic oscillator (SHO) state. Several works have studied trapped polariton condensates in the SHO state [21,22,23]. However, manipulating trapped polaritons to fully condense at the ground state of the SHO state is still a challenging work.

In this paper, we obtain quantized polariton lasing in the ground state of the SHO state using an optical potential well with strong polariton trapping. The InGaN/GaN QWs with robust localized excitons (*E_b_* = 90 meV) is utilized as the active layer of a vertical planar microcavity to achieve the robust polariton at room temperature. The k-space dispersion of polaritons is directly measured by angle-resolved photoluminescence spectra, showing a large Rabi splitting (*Ω* = 61 meV). The manipulation of polaritons through a parabolic potential trap is realized. Polaritons distribute in the SHO state when in the confinement of parabolic potential wells. The SHO state is modulated through adjusting the width of the potential well, and quantized polariton lasing is controlled to emission from the ground state of the SHO state. Our results provide a feasible route to manipulate the polariton condensation, and this scheme is also suitable for designing novel macroscopic quantum polariton devices.

**Figure 1:**
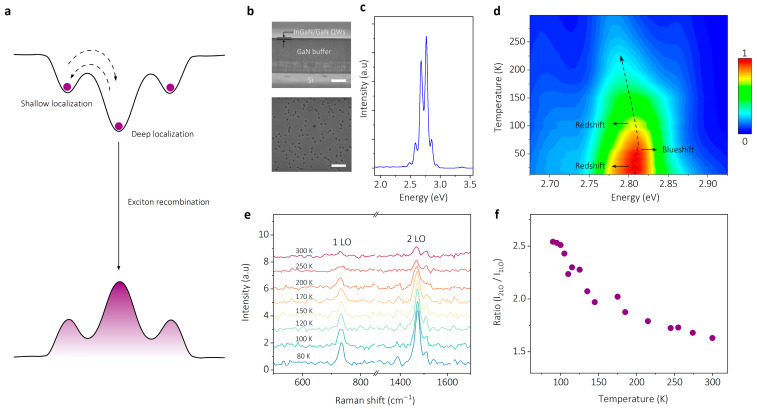
Characteristics of spatial localized excitons in InGaN/GaN quantum well (QW) samples. (**a**) Energy band diagram of localized excitons in InGaN/GaN QWs. (**b**) Scanning electron microscope images of the cross-section (top, scale bar is 1 μm) and surface morphology (bottom, scale bar is 200 nm) of the InGaN/GaN QW sample (**c**) Micro-photoluminescence (μ-PL) spectrum of the sample at room temperature (RT). (**d**) Color mapping of temperature-dependent PL spectra of the InGaN/GaN QW sample, exhibiting an S-shaped variation (as black dashed line arrow shows) in peak position as a function of temperature. The shift directions in different temperature interval are indicated by black solid arrows. (**e**) Temperature-dependent resonant Raman scattering (RRS) spectra of the sample, the first- and second-order longitudinal optical (LO) phonon scattering spikes are labeled as 1LO and 2LO. (**f**) Intensity ratio of *I_2LO_*/*I_1LO_* versus temperature.

In our experiment, six-period InGaN/GaN QWs were epitaxially grown on a Si substrate through GaN buffer layer technology. An energy band diagram of spatial localized excitons is illustrated as Figure 1a. In-N clusters similar to quantum dots are formed in InGaN/GaN QWs [24], which results in a series of spatially localized exciton states with a larger electron affinity and a smaller effective Bohr radius [25]. The intrinsic mechanism of localized excitons in InGaN alloys have been explored by S. Nakamura et al. using the scanning cathode luminescence technique [26]. The increased overlap of electron and hole wavefunction induces high exciton binding energy and high oscillator strength, and the exciton binding energy of more than 100 meV has been reported in InGaN/GaN QWs [27]. Figure 1b shows the scanning electron microscope images of the InGaN/GaN QW sample, where the top image represents the cross-section with a scale bar of 1 μm and the bottom image displays the surface morphology with a scale bar of 200 nm. Figure 1c shows the micro-photoluminescence (μ-PL) spectrum of the InGaN/GaN QW sample at room temperature. Temperature-dependent PL mapping of the sample shown in Figure 1d exhibits a typical S-shaped variation (as black dashed line arrow shows) in the PL peak position along with temperature, which has been regarded as crucial evidence of localized excitons [28,29]. In the low-temperature region (6–40 K), the peak position exhibits a normal red-shift, and the shift directions are indicated by the black solid arrows. Then, it undergoes an anomalous blueshift in the region of 40–75 K and restores the redshift at 75–300 K. The thermal dynamics process of spatial localized excitons can be explained with the diagram in Figure 1a. At low temperatures, photo-excited excitons are first to be captured by deep localized states, resulting in a red-shift of the peak position. The deep localized center exhibits the saturation of captured excitons with increasing temperature, leading to the emergence of radiation emission from nearby shallow localized states. Consequently, an abnormal blueshift phenomenon is observed for the peak position. As the temperature increases further, the peak position undergoes a red-shift due to the shrinkage of the band gap.

For further evidence of the localized exciton in InGaN/GaN QWs, temperature-dependent resonant Raman scattering (RRS) spectroscopy is performed, which has been known as an effective experimental mean for measuring the spatial localized excitons [30]. The RRS spectroscopy in Figure 1e shows two distinct peaks at 735 and 1470 cm^−1^, corresponding to the first- and second-order longitude optical phonons (1-/2-LO) of InGaN/GaN QWs (the non-resonant Raman scattering spectrum is provided in Figure S1). Notably, the intensity of the high-order 2LO peak is larger than that of the 1LO peak, indicating an abnormal phenomenon. This anomalous enhancement of *I_2LO_* intensity at low temperature is attributed to a Fröhlich interaction via the localized exciton as an intermediate electronic state [31], providing unambiguous evidence for the existence of localized excitons in InGaN/GaN QWs. As the temperature increases from 80 to 300 K, the intensity ratio of *I_2LO_*/*I_1LO_* decreases gradually (Figure 1f). Temperature dependence of the integrated PL intensity (I) is fitted by the Arrhenius model with dual activation energy (see Figure S2) to estimate the binding energy (*E_b_*) of localized excitons in InGaN/GaN QWs [32]. The best fitting yields an activation energy of approximately 90 meV, which is almost four times larger than the thermal activation energy at room temperature (~25 meV), satisfying the prerequisite for room-temperature polaritons.

**Figure 2:**
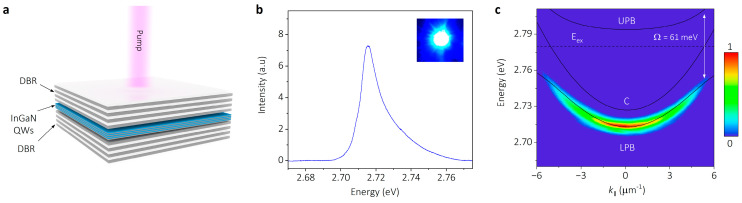
Microcavity structure and momentum space dispersion of InGaN/GaN QW microcavity polaritons at room temperature. (**a**) Schematic of planar microcavity, comprising InGaN/GaN QWs embedded between two distributed Bragg reflectors (DBRs). (**b**) Photoluminescence spectrum of InGaN/GaN QW microcavity polariton under single pump as depicted in panel a. The inset is an image of pump spot captured by a CCD camera. (**c**) Momentum space PL dispersion pattern of InGaN/GaN QW microcavity polariton measured by angle-resolved photoluminescence spectrum technology, showing a parabolic-like dispersion in k-space. The black solid lines represent theoretical fitting dispersion for cavity photon mode (C), upper polariton branch (UPB) and lower polariton branch (LPB), obtained from a rigorous two-level coupled model. The dashed black line displays the uncoupled localized exciton (*E_ex_*) dispersion. The Rabi spitting *Ω* (61 meV), calculated from the fitting dispersion, is presented in the figure.

For realizing the strong coupling between excitons and cavity photons, a vertical planar microcavity (as schematic in Figure 2a) was fabricated, which consists of two dielectric distributed Bragg reflectors (DBRs) deposited on the top and bottom of the InGaN/GaN QWs. The detail fabrication process of the microcavity is provided (see device fabrication in experimental section and supporting information in Figure S3). Figure 2b is the photoluminescence (PL) spectrum of the InGaN/GaN QW microcavity under a single pump, and the inset displays an image of the pumping spot captured by a CCD camera. The asymmetric feature of the PL peak indicates the presence of strong coupling between the exciton and cavity modes. Angle-resolved PL spectrum technology is a powerful means for directly mapping the k-space PL dispersion of polaritons. Figure 2c shows the k-space PL dispersion image of the polariton in InGaN/GaN QW microcavity, which exhibits a parabolic-like energy dispersion. In our experiment, all measurements of the polariton were carried out at room temperature. By considering the contribution of a bare exciton (*E_ex_*) and resonance cavity photon (*E_ph_*), the energy dispersion of polaritons can be rigorously calculated by solving a two-level coupled model [1]. The Hamiltonian is given by

(1) $$H=\left[\begin{array}{cc} E_{ex} & \Omega \\ \Omega & E_{ph} \end{array}\right]$$

The expression of polariton dispersion by solving (1) is obtained as follows:

(2) $$E_{LPB,UPB}\left(k_{∥}\right)=\frac{1}{2}\left[E_{ph}\left(k_{∥}\right)+E_{ex}\left(k_{∥}\right)\right]\pm \frac{1}{2}\sqrt{{\Delta E\left(k_{∥}\right)}^{2}+\Omega^{2}}$$

where $k_{∥}$ is the wave vector parallel to the in-plane of microcavity, $E_{ph}\left(k_{∥}\right)$ and $E_{ex}\left(k_{∥}\right)$ denote the energy of the cavity modes and excitons, $\Delta E\left(k_{∥}\right)$ is defined as $\Delta E\left(k_{∥}\right)$ = $E_{ph}\left(k_{∥}\right)$ − $E_{ex}\left(k_{∥}\right)$ and Ω is Rabi splitting. The energy detuning δ is defined as δ = $E_{ph}\left(k_{∥}=0\right)$ − $E_{ex}\left(k_{∥}=0\right)$, and $E_{UPB}\left(k_{∥}\right)$ and $E_{LPB}\left(k_{∥}\right)$ are the energy of the upper polariton branch (UPB) and lower polariton branch (LPB), respectively. The fitting curves of the cavity mode (C), UPB and LPB are presented in Figure 2c as black solid lines based on rigorous two-level coupled models. The localized exciton dispersion (*E_ex_* = 2.78 eV) is shown as a black dashed line. According to the best fitting, the detuning of δ = −53 meV is obtained, and the Rabi splitting is *Ω* = 61 meV. The Hopfield coefficients [33] of the LPB in Figure 2c are provided in Figure S4. The large Rabi splitting indicates the realization of the polariton through the strong coupling between excitons and cavity modes. It is challenging to observe the upper polariton branch in the k-space PL dispersion image, which is a commonly encountered situation in microcavities with large Rabi splitting due to absorption in the electron–hole continuum and rapid thermal relaxation.

**Figure 3:**
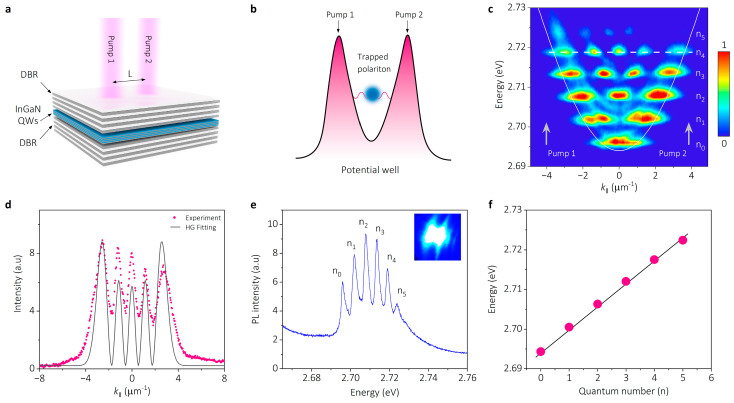
Generation of parabolic potential wells and quantized polaritons in simple harmonic oscillator (SHO) states. (**a**) Schematic of the InGaN/GaN QW microcavity with double pumping modulation. (**b**) Schematic of parabolic potential well induced by two pumping modulation and optical trapped polaritons. (**c**) The k-space PL dispersion image of optical trapped polaritons, showing a series of quantum levels (denoted as *n*_0_ to *n*_5_ from low to high energy) of the SHO state. The white solid parabola indicates the theoretical non-discretized dispersion and gray arrows show the excitation points. (**d**) Intensity distribution (circle dots) of the quantum level $\psi_{n=4}\left(x\right)$ extracted from the cross-section along white dashed line in (**c**), fitted with Hermite–Gaussian function (black curve). (**e**) PL spectrum of the trapped polaritons in the SHO state, showing six discrete energy peaks corresponding to the quantum levels in (**c**). The inset is the image of pump spot captured by a CCD camera. (**f**) The peak energies (circle dots) extracted from (**e**) as a function of quantum number (*n*), fitted by energy level formula $E=(n+\frac{1}{2})\hbar \omega$ of a standard SHO state.

In the planar microcavity, polaritons are a confined state in the direction perpendicular to the microcavity plane, but they exist in an extended state within the microcavity plane [34]. By incorporating additional lateral traps within the microcavity plane, it becomes feasible to create a tailored potential well that modifies the interactions and transport behavior of polaritons, thereby facilitating trapped polariton condensation [15]. As depicted in Figure 3a, optical pump control is used to manipulate polariton states in our experiment. The schematic shown in Figure 3b illustrates that high-density polariton reservoirs are formed around the two pump points to create potential barriers, and these specific potential barriers form a parabolic potential well. Polaritons in the reservoirs undergo an outward force due to a strong repulsive interaction between polaritons, projecting into the parabolic potential well. Simultaneously, the polaritons trapped by the potential well are redistributed in both energy and space to occupy simple harmonic oscillator (SHO) states [21]. The k-space PL dispersion of the trapped polariton state in Figure 3c presents a series of typical quantum levels of the SHO state, which are denoted as *n*_0_ to *n*_5_ from low to high energy, and the energy spacing among quantum levels is equal (5.3 meV). The white solid parabola indicates the theoretical non-discretized dispersion, and gray arrows show the position of the pump points.

Along the horizontal white dashed line in Figure 3c, the intensity distribution of the quantum level *n* = 4 is extracted as circle dots in Figure 3d, which is fitted by the Hermite–Gaussian wave function:

(3) $$\psi_{n}(\xi )={\left(\frac{\alpha }{\sqrt{\pi }2\pi n!}\right)}^{1/2}H_{n}\left(\xi \right)e^{-\xi^{2}/2}$$

where *α* and *H_n_*(*ξ*) are the normal constant and Hermite polynomials, respectively, and n is the energy level number. The experiment data are fitted well with the Hermite–Gaussian wave function, indicating the characteristic of stable quantum oscillator wavefunctions of the SHO state in k-space. The PL spectrum of the trapped polariton is shown in Figure 3e, presenting six discrete peaks which correspond to the quantum levels in Figure 3c. The inset is the pump spot image captured by the CCD camera. The peak energies (extracted from Figure 3e) as a function of the quantum number (*n*) are summarized in Figure 3f (circle dots), which is fitted by a standard energy level formula of SHO states: $E=(n+\frac{1}{2})\hbar \omega$, where n represents the quantum number of the wave function, and ℏω is the energy spacing between levels. And, the fitting in Figure 3f shows a linear relationship between the energy level and quantum number, further verifying the simple harmonic oscillator state of the trapped polaritons.

**Figure 4:**
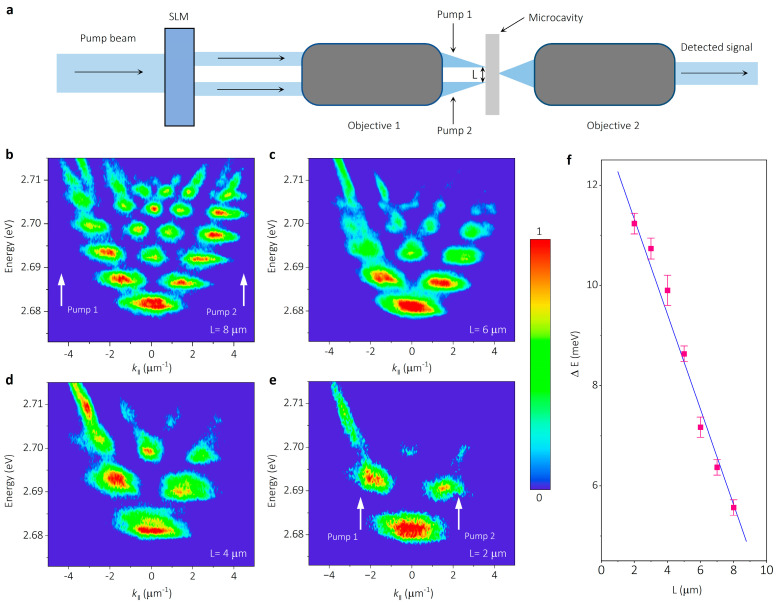
Manipulation of the SHO state in parabolic potential wells with different widths. (**a**) The schematic of experimental setup for controlling the width of parabolic potential wells, where the width (*L*) is controlled by adjusting the spacing of the pumping spot modulated through the digital pattern put into a spatial light modulator (SLM). (**b**) PL dispersion image of the SHO state with a width of *L* = 8 μm, and up to six energy levels of the SHO state are observed. The white arrows show the position of pump 1 and pump 2. (**c**) As the potential width decreases to *L* = 6 μm, reduction in energy levels is shown. (**d**) Narrowing the potential well to *L* = 4 μm, a more sparse distribution of the quantized energy levels can be seen. (**e**) At a width of *L* = 2 μm, the quantization becomes highly discrete, with few of prominent energy levels remaining. (**f**) The energy spacing (ΔE) between SHO sublevels *n*_0_ and *n*_1_ as a function of the potential trap width (*L*). The plot clearly shows an inverse proportional relationship between energy spacing and potential width.

The width (*L*) of the potential well can be controlled by adjusting the spacing of the pumping spots to thus manipulate the quantum levels of the SHO state flexibly. As shown in the schematic of the experimental setup (Figure 4a), a spatial light modulator (SLM), known as a powerful tool for making the pump beam to specific patterns, is used in our experiment. The width of the potential well is controlled through adjusting the spacing of the pumping spot modulated by the digital patterns put into the SLM. When the potential width is *L* = 8 μm, up to six energy levels are observed in Figure 4b, and the white arrows display the pump positions. With the width decreasing from 6 μm to 2 μm, the amounts of quantized polariton energy levels reduce gradually. Meanwhile, the energy spacing of the SHO state increases moderately, the evolution of which is clearly observed from the k-space PL dispersion in Figure 4c–e. When the height of the potential well is fixed, the energy spacing between the SHO states can be given as

(4) $$\hbar \omega \propto \frac{\hbar }{L}\sqrt{\frac{1}{m^{*}}}$$

where ℏω is the energy spacing, $m^{*}$ is the effective mass of the polaritons, L is the potential width. To eliminate the influence of varying effective mass at different energy, we extract the energy spacing from the same energy position. As shown in Figure 4f, the energy spacing between ground state *n*_0_ and first excited state *n*_1_ is plotted as a function of potential width (red square dots). Evidently, level spacing ΔE depends inversely on the potential width (as the blue straight linear line shows), which are consistent with the relationship of the energy spacing and potential width in SHO theory. Additionally, the maximum energy spacing of 11.3 meV is realized between the SHO sublevels by using a potential well with a width of 2 μm, indicating the robust polariton trapping of the optical parabolic potential well in our experiment.

**Figure 5:**
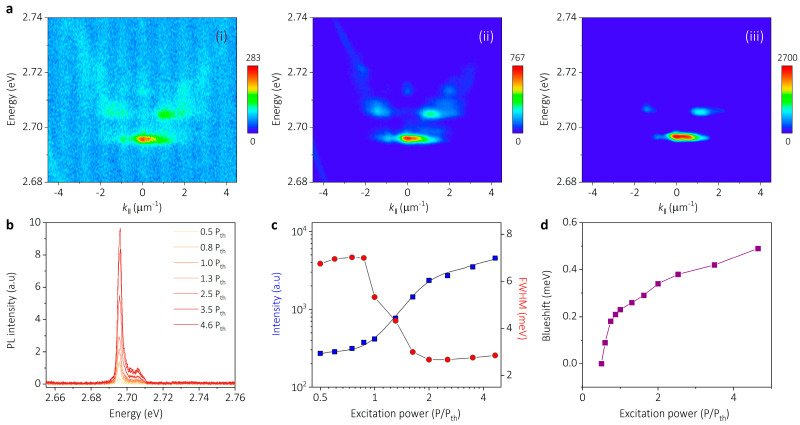
Characteristic of quantized polariton lasing in the SHO state. (**a**) Power-dependent k-space PL dispersion of the SHO state trapped by a 2 μm width potential well at 0.8 P_th_, 1.3 P_th_, and 2.5 P_th_ (panels (**i**–**iii**), respectively). P_th_ (65 μJ cm^−2^) is the excitation power of the condensation threshold. Above the threshold, the ground state of the SHO state is massively occupied by polaritons and exhibits significantly enhanced emission intensity, indicating the onset of polariton lasing. (**b**) PL spectra of the SHO state at *k*_‖_ = 0. With excitation power increasing, a sharp increase in PL intensity is observed beyond the threshold. (**c**) Log–log plot of the PL intensity of the SHO state at *k*_‖_ = 0 and the full width at half-maximum (FWHM) versus excitation power. The nonlinear increase in the intensity along with the narrowing of the linewidth indicate the occurrence of polariton lasing. (**d**) Blueshift of the polariton emission versus excitation power, showing an inflection point around the condensation threshold.

Coherent quantized microcavity polariton lasing can be obtained by controlling the trapped polaritons to condense in the ground state of the SHO state. As the above result shows, a 2 μm width potential well has a robust trapping ability for confining the condensates into the ground state of the SHO. The characteristic of the quantized polariton lasing in the 2 μm width potential well is systematically investigated. Figure 5a(i–iii) present the power-dependent k-space PL images of the trapped polaritons, the excitation powers of which are given as 0.8, 1.3 and 2.5 P_th_, where P_th_ (65 μJ cm^−2^) is the excitation power of the condensation threshold. At a low excitation power (0.8 P_th_), the trapped polaritons exhibit a comparable broad distribution in the SHO state. As the excitation power increases to 1.3 P_th_, the emission intensity of the trapped polariton increases greatly. Meanwhile, the polariton distribution shrinks to a smaller area. Under a higher excitation power (2.5 P_th_), the trapped polaritons fully condense at the ground state of the SHO, realizing quantized polariton lasing.

The PL spectra of the SHO state at *k*_‖_ = 0 display a nonlinear increasing of PL intensity and narrowing of the linewidth with the excitation power increasing (Figure 5b). To characterize this transition quantitatively, we plot the PL intensity of the SHO state at *k*_‖_ = 0 as a function of the excitation power in a log–log scale (square dots in Figure 5c). As the excitation power increases above the threshold (65 μJ cm^−2^), the output PL intensity of the SHO state presents a clear nonlinear increase by one order of magnitude. Meanwhile, the linewidth of the emission peak narrows from 7.0 to 3.0 meV upon further increasing the excitation power to 1.7 P_th_, indicating a spontaneous building up of the temporal coherence in the condensation regime. The energy blueshift of the polariton emission versus the excitation power is plotted in Figure 5d. The observed continuous blueshift of the polariton emission provides important evidence for the polariton condensation in the SHO state. The slope of the energy blueshift notably exhibits a flex point near the threshold, which signifies two distinct interaction mechanisms contributing to the energy blueshift. It has been proposed that the interaction between polaritons and the reservoir of excited states is dominant below the threshold and that the polariton–polariton interaction will dominant the blueshift above the threshold. The disparity in strength between these two mechanisms gives rise to these two distinctive slopes of the blueshift [35].

**Figure 6:**
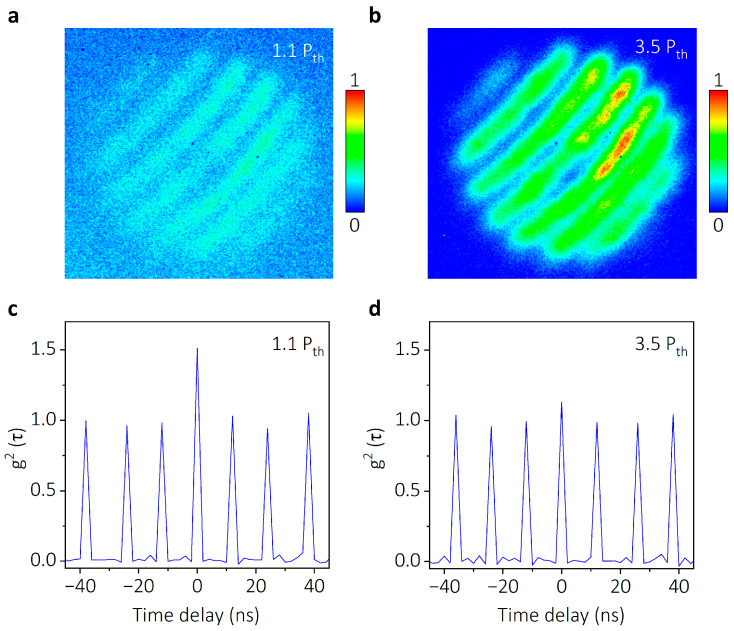
Coherence property measurement of quantized polariton condensation lasing. (**a**,**b**) Interference fringes of quantized polariton lasing measured by Michelson interferometers. At an excitation power of 1.1 P_th_, a fringe visibility of 34% is shown. As the excitation increases to 3.5 P_th_, the fringe visibility is elevated to 71%, showing an increased spatial coherence of the quantized polariton lasing. (**c**,**d**) Second-order correlation function g^(2)^(τ) of the quantized polariton lasing measured by Hanbury Brown–Twiss interferometry. At an excitation power of 1.1 P_th_, a g^(2)^(0) of 1.43 is displayed. As the excitation increases to 3.5 P_th_, the g^(2)^(0) is obtained as 1.14. The decrease in g^(2)^(0) supports the quantum phase transition of exciton–polariton systems from a classical thermal state to a condensed coherent state.

To further characterize the coherence properties of quantized polariton condensates in the SHO state, the quantized polariton lasing emission is analyzed by a Michaelson interferometer and Hanbury Brown–Twiss (HBT) interferometry, respectively. The interference patterns of the polariton lasing measured by Michaelson interferometers are shown in Figure 6a,b. At an excitation power of 1.1 P_th_, a fringe visibility of 34% is presented, where fringe visibility is defined as $\upsilon =(I_{max}-I_{min})/(I_{max}+I_{min})$, and $I_{max(min)}$ is the maximum (minimum) intensity of the fringes, respectively. With the excitation power increasing to 3.5 P_th,_ the fringe visibility is elevated to 71%. The increase in fringe visibility indicates the enhanced spatial coherence of the polariton lasing, supporting the formation of polariton condensates. The second-order correlation function g^(2)^(τ), where τ is the delay time, is served as a crucial criterion for characterizing the quantum coherent state, and it has been extensively employed to unveil the quantum coherence properties of polaritons [10,36]. The g^(2)^(τ) values of the polariton lasing emission measured by Hanbury Brown–Twiss (HBT) interferometry are displayed in Figure 6c,d. At an excitation power of 1.1 P_th_, a g^(2)^(0) of 1.43 is shown. As the excitation increases to 3.5 P_th_, the g^(2)^(0) decreased to 1.14, as observed in the plot. According to the theory of photon coincidence counting, the g^(2)^(0) of a classical thermal state is expected to be 2. As for a quantum coherent state (quantum mechanical pure state), g^(2)^(0) = 1. The g^(2)^(0) of the quantized polariton lasing decreased from 1.43 to 1.14 as the excitation power increased from 1.1 P_th_ from 3.5 P_th_, which supported the quantum phase transition of the exciton–polariton systems from a classical thermal state to a condensed coherent state [37,38,39]. Thus, the well coherence property of the quantized polariton lasing is confirmed by the measurements of spatial interference patterns and g^(2)^(τ).

In conclusion, room-temperature quantized polariton lasing is realized in InGaN/GaN QW planar microcavity. Due to the strong localized excitons in InGaN/GaN QWs, room-temperature polaritons with large Rabi splitting (61 meV) are obtained under a strong coupling regime. The parabolic potential well induced by the optical pump control is used to manipulate the trapped polariton condensates to occupy the SHO state. It demonstrates that the number of quantum levels of the SHO state are well modified by adjusting the width of the potential well. The quantized microcavity polariton lasing with well coherence is feasible to realize by controlling the polaritons so that they condense in the ground state of the SHO state using a potential well that has a width of 2 μm. Our results offer a feasible route to the manipulation of polariton condensates and engineering polariton quantum simulators and ultralow threshold lasers.

## 2. Experimental Section

Growth of InGaN quantum wells (QWs): A host InGaN/GaN QW active layer for 2D spatial localized excitons was grown on Si (111) substrate by a metalorganic chemical vapor deposition (MOCVD) system. Trimethylgallium (TMGa), trimethylaluminum (TMAl) and ammonia (NH_3_) were employed as the reactant source materials for Ga, Al and N, respectively. H_2_ was used as the carrier gas for the growth of HT-AlN, compositionally graded AlGaN and GaN. The cleaned Si (111) substrates were immersed in H_2_SO_4_:H_2_O_2_ (4:1) solution for 5 min and etched with HF (10%) for 1 min to remove the surface oxide layer. Before introducing NH_3_ into the reactor, aluminum was pre-deposited on the Si substrates for 5 s to prevent the formation of SiN_x_. Next, a 100 nm AlN was deposited as the seeding layer, then a 2 μm thick GaN buffer layer was grown. Finally, six-period InGaN (3 nm)/GaN (10 nm) QWs were grown on the GaN layer.

Device fabrication: Firstly, the bottom distributed Bragg reflectors (DBRs) made of 10 HfO_2_/SiO_2_ pairs were deposited on the as-grown InGaN/GaN QW sample by using e-beam evaporation. Then, the sample was bonded to a sapphire, and Si substrate was removed by selective wet etching. Next, the GaN buffer layer was specially polished to remove the substrate. Subsequently, the top DBRs made of 8 HfO_2_/SiO_2_ pairs were deposited on the polished surface. Consequently, the vertical planar microcavity embedded with the InGaN/GaN QW active layer was fabricated.

Optical Characterization: A micro-photoluminescence system (Horiba HR; Northampton, UK) with a CW He-Cd laser (325 nm), which has a linewidth of 0.3 nm and radiation power of 50 mW, was used to perform the measurement of resonant Raman scattering and PL spectroscopy for the InGaN/GaN QW sample. Temperature-dependent PL spectra were carried out by using a closed-cycle liquid helium cryostat (Advanced Research Systems). The PL signal was collected by a 0.5 m monochromator (Omni-λ500) with 1200 g/mm grating and detected with a lock-in amplifier photomultiplier tube. The k-space dispersion of the microcavity of the polariton was measured by self-constructed angle-resolved μ PL system with an NUV objective (50×, 0.42 N.A.). The InGaN/GaN QW microcavity was excited by using a mode-locked Ti-Sapphire fs pulse laser with a duration of 500 fs and a repetition rate of 80 MHz, centered at a wavelength of 400 nm. The excitation pulse with 100 μJ cm^−2^ was used to pump the InGaN/GaN QW microcavity unless there were specific notes. The interference fringes were recorded by a Michelson interferometer.

HBT Interferometer: The optical signal from the microcavity was filtered by a monochromator and separated into two paths via a 50:50 fiber beam splitter. The photons from two fibers were recorded using a pair of single-photon detectors, and their statistical properties (g^(2)^ functions) were measured by a time-correlated single-photon counting module.

## Data Availability

The original contributions presented in the study are included in the article/Supplementary Material, further inquiries can be directed to the corresponding authors.

## Supplementary Materials

The following supporting information can be downloaded at: https://www.mdpi.com/article/10.3390/nano14141197/s1, Figure S1: The non-resonant Raman scattering spectrum of the InGaN QW sample at room temperature; Figure S2: Temperature-dependent integrated PL intensity of the InGaN/GaN QWs. The solid curve represents the best fit with Arrhenius model, yielding a binding energy of 90 meV; Figure S3: The detail fabrication process of InGaN/GaN QW planar microcavity; Figure S4: The Hopfield coefficients of the LPB for localization exciton polariton in the microcavity.
