# Supplementary material for: Quantized Microcavity Polariton Lasing Based on InGaN Localized Excitons
Source: Nanomaterials (Basel). 2024 Jul 14;14(14):1197. doi: 10.3390/nano14141197 (PMC11279400; doi:10.3390/nano14141197)
Supplement: Supplementary file 1 [file nanomaterials-14-01197-s001.zip › nanomaterials-3085080-supplementary.pdf]

nanomaterials

Supporting Information

Quantized Microcavity Polariton Lasing Based on InGaN Localized Excitons

Huying Zheng [1], Runchen Wang [1], Xuebing Gong [1], Junxing Dong [1], Lisheng Wang [1], Jingzhuo Wang [1], Yifan Zhang [1], Yan Shen [2,*], Huanjun Chen [2], Baijun Zhang [2,*] and Hai Zhu [1,*]

1 State Key Laboratory of Optoelectronic Materials and Technologies, School of Physics, Sun Yat-sen University, Guangzhou 510275, China; zhenghy37@mail2.sysu.edu.cn (H.Z.); wangrch7@mail2.sysu.edu.cn (R.W.); gongxb@mail2.sysu.edu.cn (X.G.); dongjx3@mail2.sysu.edu.cn (J.D.); wanglsh23@mail2.sysu.edu.cn (L.W.); wangjzh23@mail2.sysu.edu.cn (J.W.); zhangyf93@mail2.sysu.edu.cn (Y.Z.)
2 State Key Laboratory of Optoelectronic Materials and Technologies, School of Electronics and Information Technology, Sun Yat-sen University, Guangzhou 510275, China; chenhj8@mail.sysu.edu.cn
* Correspondence: shenyan7@mail.sysu.edu.cn (Y.S.); zhbaij@mail.sysu.edu.cn (B.Z.); zhuhai5@mail.sysu.edu.cn (H.Z.)

The non-resonant Raman spectrum of InGaN QWs sample excitated by a 633 nm laser is presented in Figure S1. The peaks at 735, 566, 556, 531, 143 cm^{-1} correspond to the phonon modes of A_1(LO), E_2(high), E_1(TO), A_1(TO), and E_2 (low) respectively.

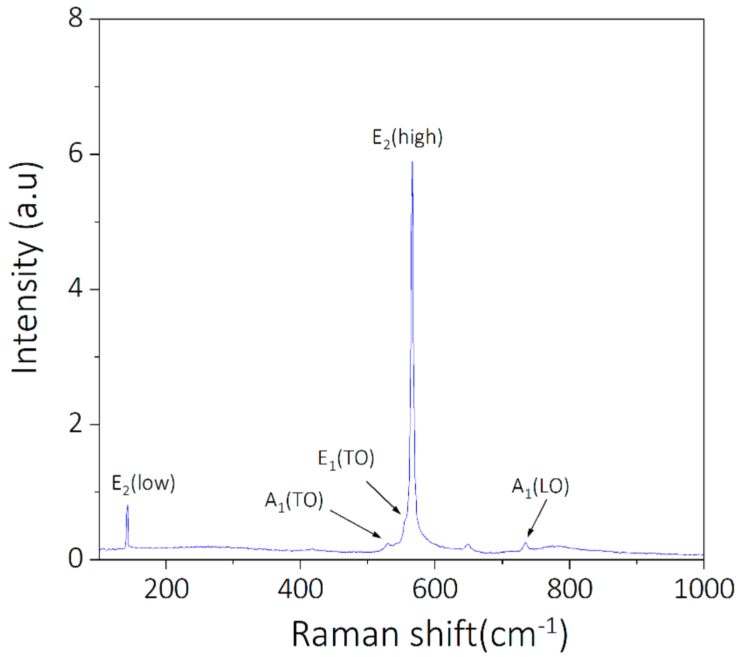

Figure S1. The non-resonant Raman scattering spectrum of the InGaN QWs sample at room-temperature.

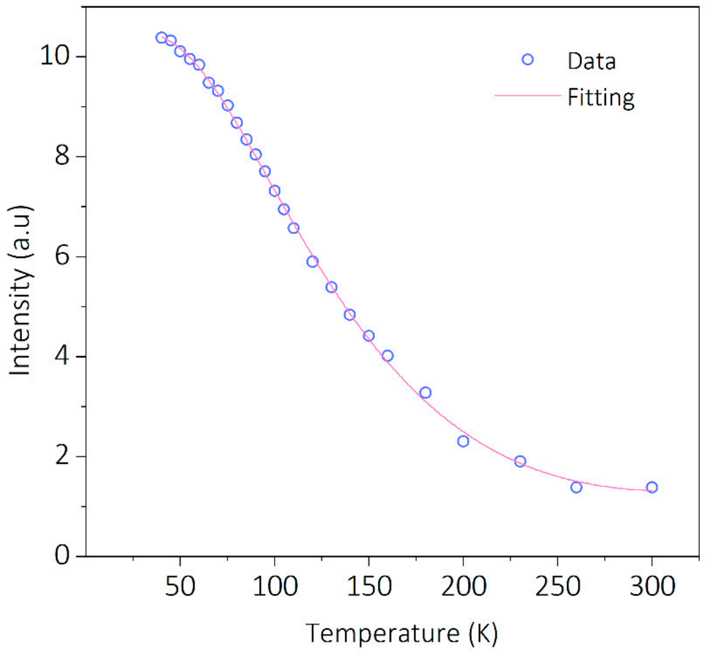

Figure S2. Temperature-dependent integrated PL intensity of the InGaN/GaN QWs. The solid curve represents the best fit with Arrhenius model, yielding a binding energy of 90 meV.

The thermal activation energy of localized exciton can be well described by the Arrhenius model with dual activation energy:

$$I(T) = \frac{I_0}{C_1 exp(-E_1/k_B T) + C_2\, exp(-E_2/k_B T) + 1} \quad \text{(S1)}$$

where E_1 and E_2 represent the thermal activation energies of two different processes, and C_1 and C_2 are parameters indicating the relative ratios of nonradiative recombination. $I(T)$ and I_0 are the integrated PL intensity. As presented in Figure S2, the temperature-dependent integrated PL intensity of InGaN/GaN QWs is fitted using the formula of Arrhenius model in S1. The solid curve represents the best fit giving the activation energies of E_1= 22 meV and E_2 = 90 meV. Activation energy E_1 is related to the thermally activated capture of localized excitons by nonradiative defects in barrier layer, while E_2 corresponds to the thermal activation energy required for the dissociation of localized exciton into continuum states. Therefore, the activation energy E_2 can be regarded as the binding energy of the localized excitons in InGaN/GaN QWs. The binding energy of our InGaN/GaN QWs (90 meV) is nearly four times the thermal activation energy at room-temperature (~25 meV), which fully satisfies the required conditions for producing room-temperature polaritons.

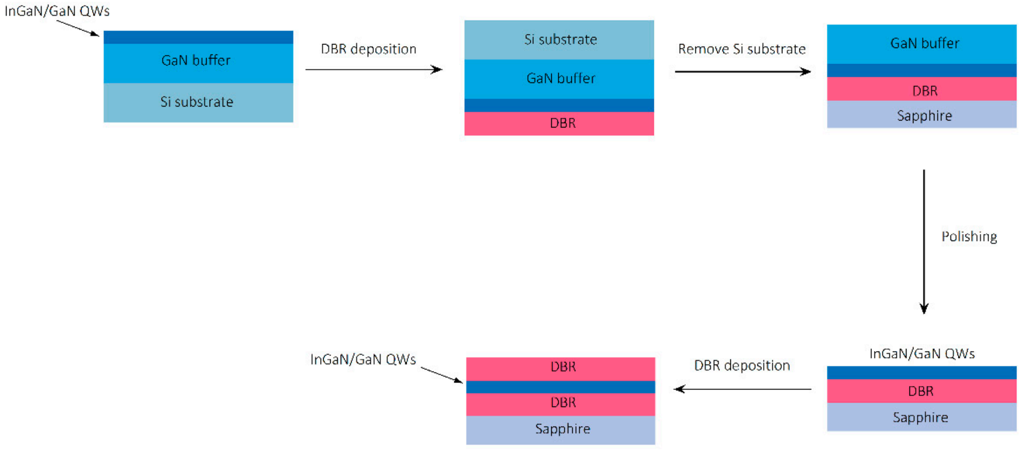

Figure S3. The detail fabrication process of InGaN/GaN QWs planar microcavity.

Figure S3 illustrates the fabrication process of the InGaN/GaN QWs planar microcavity. Firstly, six-periods InGaN/GaN QWs was epitaxially grown on Si substrate through a GaN buffer layer technology. Subsequently, the bottom distributed Bragg reflectors (DBRs) made of 10 HfO2/SiO2 pairs was deposited on the as-grown InGaN/GaN QWs sample using e-beam evaporation. Then, the sample was bonded on a sapphire template, and Si substrate was removed by selective wet etching. Next, the GaN buffer layer was polished, and the top DBR made of 8 HfO2/SiO2 pairs was deposited. Finally, the vertical planar microcavity with InGaN/GaN QWs as the active layer was fabricated.

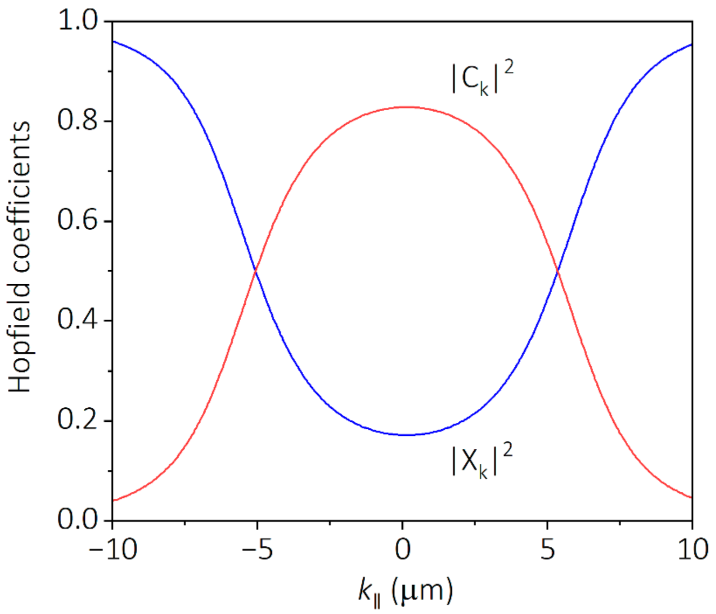

Figure S4. The Hopfied coefficients of the LPB for localization exciton polariton in the microcavity.

As proposed by Hopfield, photon and excition fraction in polariton branch can be given by the amplitude of $C_{k_{\parallel}}$ and $X_{k_{\parallel}}$ which are referred to as the Hopfield coefficients. The expressions are as follows:

$$\left|X_{k_{\parallel}}\right|^2 = \frac{1}{2}\left(1 + \frac{\Delta E(k_{\parallel})}{\sqrt{\Delta E(k_{\parallel})^2 + \Omega^2}}\right), \quad \left|C_{k_{\parallel}}\right|^2 = \frac{1}{2}\left(1 - \frac{\Delta E(k_{\parallel})}{\sqrt{\Delta E(k_{\parallel})^2 + \Omega^2}}\right) \tag{S2}$$

The Hopfield coefficients satisfy $\left|X_{k_{\parallel}}\right|^2 + \left|C_{k_{\parallel}}\right|^2 = 1$. Figure S4 plots the Hopfield coefficients of LPB at Fig. 2c , showing that the photon and exciton fraction at $k_{\parallel}$= 0 are $\left|C_{k_{\parallel}}\right|^2$= 0.83, $\left|X_{k_{\parallel}}\right|^2$= 0.17.
